# Parathyroid autotransplantation into the brachioradialis protects parathyroid function during total thyroidectomy

**DOI:** 10.3389/fendo.2026.1852443

**Published:** 2026-08-18

**Authors:** Shuang Wang, Yingming Liu, Gang Wu, Yuhan Gao, Huadong Qin, Chenlei Shi

**Affiliations:** The Fourth Department of General Surgery, Second Affiliated Hospital, Harbin Medical University, Harbin, China

**Keywords:** autotransplantation, brachioradialis, parathyroid, surgery, survival

## Abstract

**Background:**

Surgery remains the foundation of differentiated thyroid cancer management. However, total thyroidectomy (TT) with lymph node dissection frequently predisposes patients to hypoparathyroidism (HypoPT). The sternocleidomastoid is the conventional site for parathyroid autotransplantation, but this approach cannot effectively assess graft viability and real-time functional monitoring.

**Methods:**

Two sequential experiments were conducted. In Experiment 1, graft survival criteria were established by analyzing serum parathyroid hormone (PTH) ratios in the bilateral cephalic veins among 30 controls and 100 patients undergoing brachioradialis parathyroid autotransplantation. In Experiment 2, 180 TT patients undergoing bilateral central neck dissection were allocated into three groups (60 per group): *in situ* preservation, brachioradialis, or sternocleidomastoid autotransplantation. To address confounding, propensity score matching (PSM, 1:1:1) was performed, yielding 50 well-balanced patients per group. Temporary and permanent HypoPT, and validated graft survival were evaluated.

**Results:**

In Experiment 1, bilateral PTH ratio ≤ 1.16 was defined as the physiological baseline, with ≥ 1.2-fold as the graft viability threshold, which showed superior 6-month survival vs the 1.5-fold standard (83% vs 74%, *p* < 0.05). Graft weight predicted survival (AUC = 0.654, cutoff = 28.5 mg). The brachioradialis group showed a significantly lower incidence of temporary postoperative HypoPT than the *in situ* preservation and sternocleidomastoid groups on day 1 and day 3, and than the *in situ* preservation group on week 1 (all *p* < 0.05). Permanent HypoPT rates were comparable across groups (*p* > 0.05). Under the 1.2-fold criterion, brachioradialis graft survival rates were significantly higher than those at 1.5-fold criterion on day 1, 3 and week 1 (all *p* < 0.05). These findings were confirmed by PSM analysis.

**Conclusions:**

Brachioradialis parathyroid autotransplantation is safe and effective, while significantly reducing the risk of early postoperative HypoPT. Brachioradialis autotransplantation were recommended for compromised or inadvertently excised parathyroid glands.

**Clinical trial registration:**

https://www.chictr.org.cn/bin/home, identifier ChiCTR2400080970.

## 1 Introduction

Surgery remains the primary therapeutic modality for differentiated thyroid cancer. Complete resection of the primary tumor and involved adjacent tissues, combined with systematic lymph node dissection, reduces the risk of recurrence and metastasis. Total thyroidectomy (TT) not only improves survival and decreases recurrence risk while minimizing residual disease, but also facilitates postoperative radioactive iodine therapy and dynamic monitoring of serum thyroglobulin (Tg) levels (1, 2).

Hypoparathyroidism (HypoPT) and hypocalcemia are well-documented complications of TT that significantly compromise postoperative quality of life (3). Current parathyroid-preserving strategies encompass meticulous capsular dissection techniques (4), intrathyroidal injection of tracers, such as nanocarbon or mitoxantrone, to facilitate parathyroid identification through negative imaging (5, 6), and autotransplantation of glands with compromised *in situ* vascularity or inadvertently excised (7). The sternocleidomastoid is the conventional recipient site for parathyroid autotransplantation (8); however, this approach precludes reliable assessment of graft viability and real-time monitoring of functional status. This study aimed to investigate the survival outcomes and establish viability criteria for parathyroid autotransplantation into the brachioradialis, and to evaluate its efficacy to reduce the risk of HypoPT following TT.

## 2 Materials and methods

### 2.1 Patient selection

The cohort of Experiment 1 consisted of 30 controls (no history of thyroid surgery) and 100 consecutive patients who underwent total thyroidectomy with brachioradialis parathyroid autotransplantation at the Department of Thyroid Surgery of the Second Affiliated Hospital of Harbin Medical University between February 2024 and February 2025. The cohort of Experiment 2 included 180 patients following TT combined with bilateral central neck dissection (CND) allocated into three groups (n = 60 each) based on intraoperative parathyroid management: *in situ* preservation (ISP), sternocleidomastoid autotransplantation (SAT), or brachioradialis autotransplantation (BAT). Group allocation to the SAT or BAT group was based on patient preference after comprehensive preoperative counseling regarding the benefits and limitations of each transplantation site. All participants were followed for 6 months postoperatively. The inclusion criteria were (1): No history of head and neck surgery or radiation therapy (2); Signed informed consent by patients/guardians (3); Preoperative serum parathyroid hormone (PTH) and calcium concentrations within normal limits; and (4) Transplantation of only one parathyroid gland (PTG) intraoperatively, with postoperative pathology confirming no inadvertently excised parathyroid tissue. This study was approved by the Institutional Review Board of Second Affiliated Hospital of Harbin Medical University (KY2023-169) and was registered at Chinese Clinical Trial Registry (ChiCTR) (Registration No.: ChiCTR2400080970). Written informed consent was obtained from all participants.

### 2.2 Transplantation method

All surgical procedures were performed by a single senior surgeon. The transplanted PTGs were positioned in either the right brachioradialis or the ipsilateral sternocleidomastoid. Blood sampling of all patients was uniformly performed from the cephalic vein at the elbow level.

#### 2.2.1 Identification of PTGs

**Figure 1:**
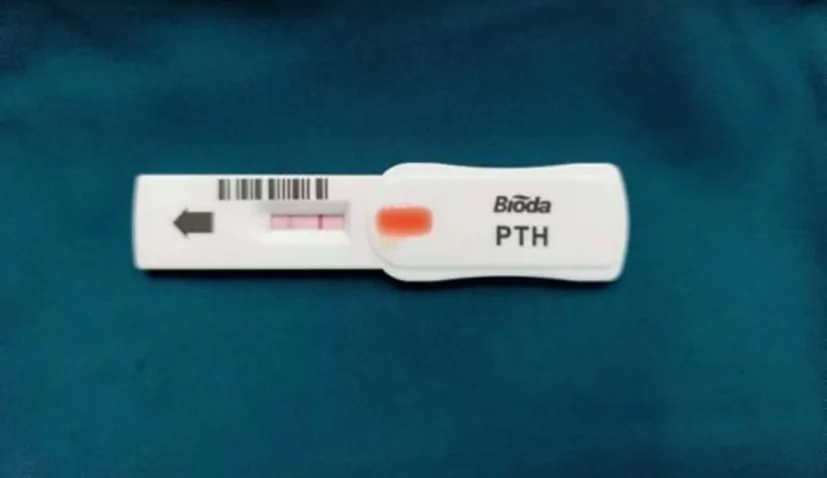
Identification of PTG tissue via colloidal gold assay.

During thyroidectomy, carbon nanoparticles were used as an adjunct negative imaging tracer for PTG identification. All grafts were further verified by colloidal gold assay to confirm parathyroid tissue (**Figure 1**). Parathyroid autotransplantation was performed when a gland exhibited dark purple discoloration, flaccid consistency, or confirmed transection of the vascular pedicle. The affected gland was gently grasped with fine forceps and sharply dissected from the thyroid capsule using ophthalmic scissors to ensure intact removal. If inadvertently excised *en bloc* with the thyroid specimen, the PTG was promptly identified and separated.

#### 2.2.2 Parathyroid transplantation procedure

##### 2.2.2.1 Graft tissue preparation

**Figure 2:**
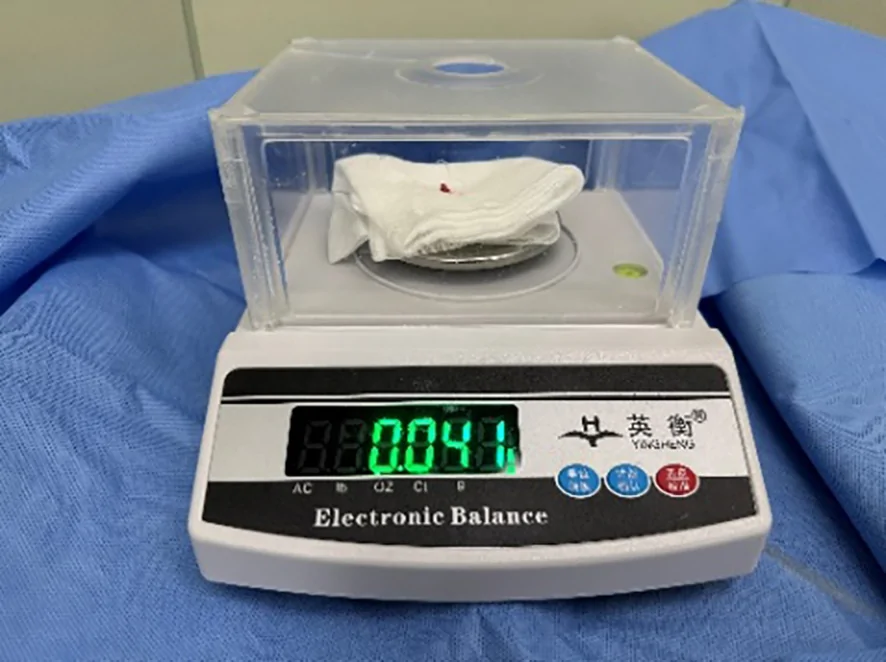
Autologous parathyroid tissue.

Following histological confirmation, adherent adipose and fibrous connective tissues were meticulously excised using a scalpel blade or ophthalmic scissors. The PTG was gently irrigated with normal saline to remove residual surface debris, and excess moisture was absorbed with sterile gauze. The specimen was then precisely weighed using an electronic microbalance (precision, 0.001 g) and the data recorded (**Figure 2**). The purified parathyroid tissue was placed in a specimen cup, minced into fragments smaller than 1 mm, and mixed with normal saline. The tissue suspension was aspirated into a 1-mL syringe fitted with a needle typically used for 20-mL syringes in preparation for autotransplantation.

##### 2.2.2.2 Graft tissue injection

**Figure 3:**
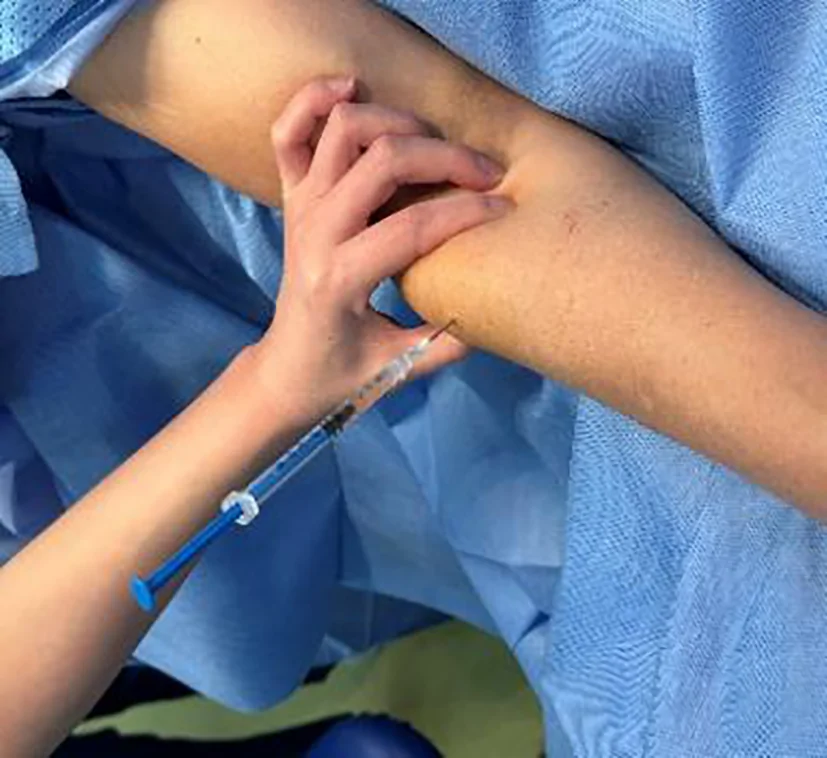
Autologous parathyroid transplantation into the brachioradialis.

**Figure 4:**
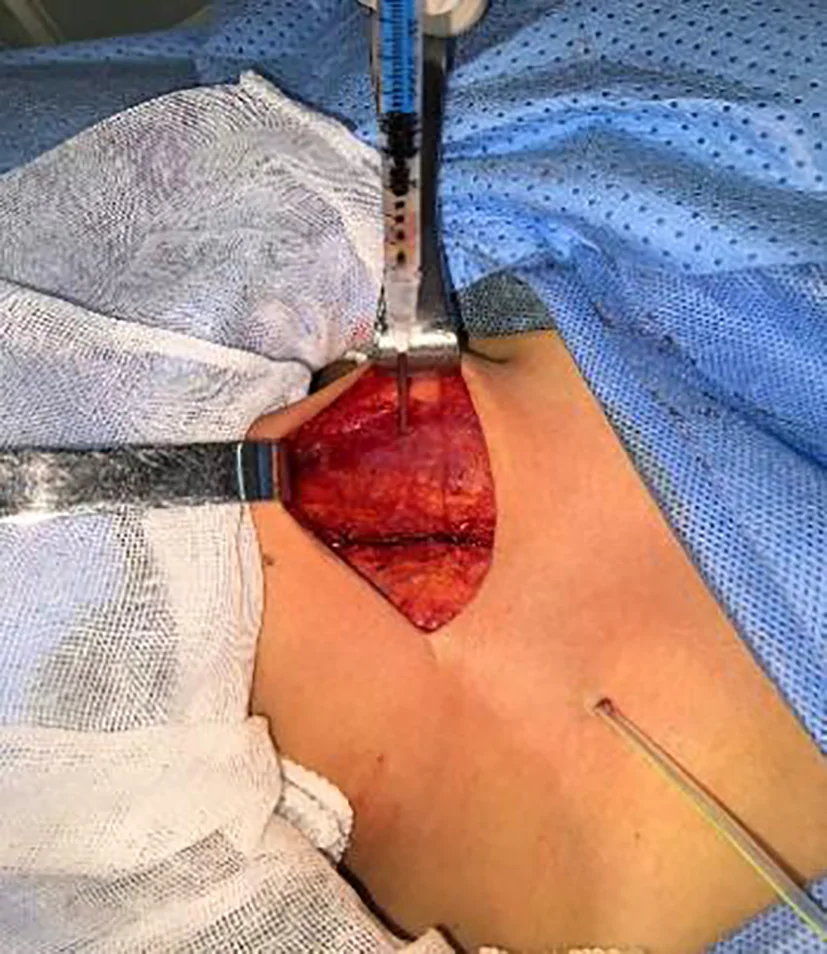
Autotransplantation of PTGs into the sternocleidomastoid.

For brachioradialis transplantation, the skin was pinched between the index finger and thumb at a site approximately 5 cm from the elbow to ensure intramuscular placement of the needle tip. Following negative blood aspiration, the prepared tissue suspension was injected into the brachioradialis (**Figure 3**). The syringe and needle were subsequently flushed with approximately 0.2 mL of normal saline to deliver any residual parathyroid tissue into the muscle. For sternocleidomastoid transplantation, the identical injection and irrigation protocol was performed under direct visualization (**Figure 4**).

### 2.3 Test index

The following patient parameters were recorded: medical record number, name, sex, age, date of surgery, surgical procedure, diabetes/hypertension status, preoperative serum calcium, PTH, 25-hydroxyvitamin D3 (25-OH-D3), Tg, thyroglobulin antibody (TgAb), hemoglobin and albumin concentrations, body mass index (BMI), tumor location, tumor size, tumor stage, extrathyroidal extension status, number of central compartment lymph nodes dissected, central lymph node metastasis, and occurrence of temporary or permanent HypoPT. In Experiment 1, PTG weight was measured prior to transplantation.

#### 2.3.1 Experiment 1

Serum PTH and calcium concentrations were measured from the cephalic veins at the elbow on postoperative day (POD) 1, POD 3, postoperative week (POW) 1, POW 2, postoperative month (POM) 1, POM 3, and POM 6.

#### 2.3.2 Experiment 2

**Figure 5:**
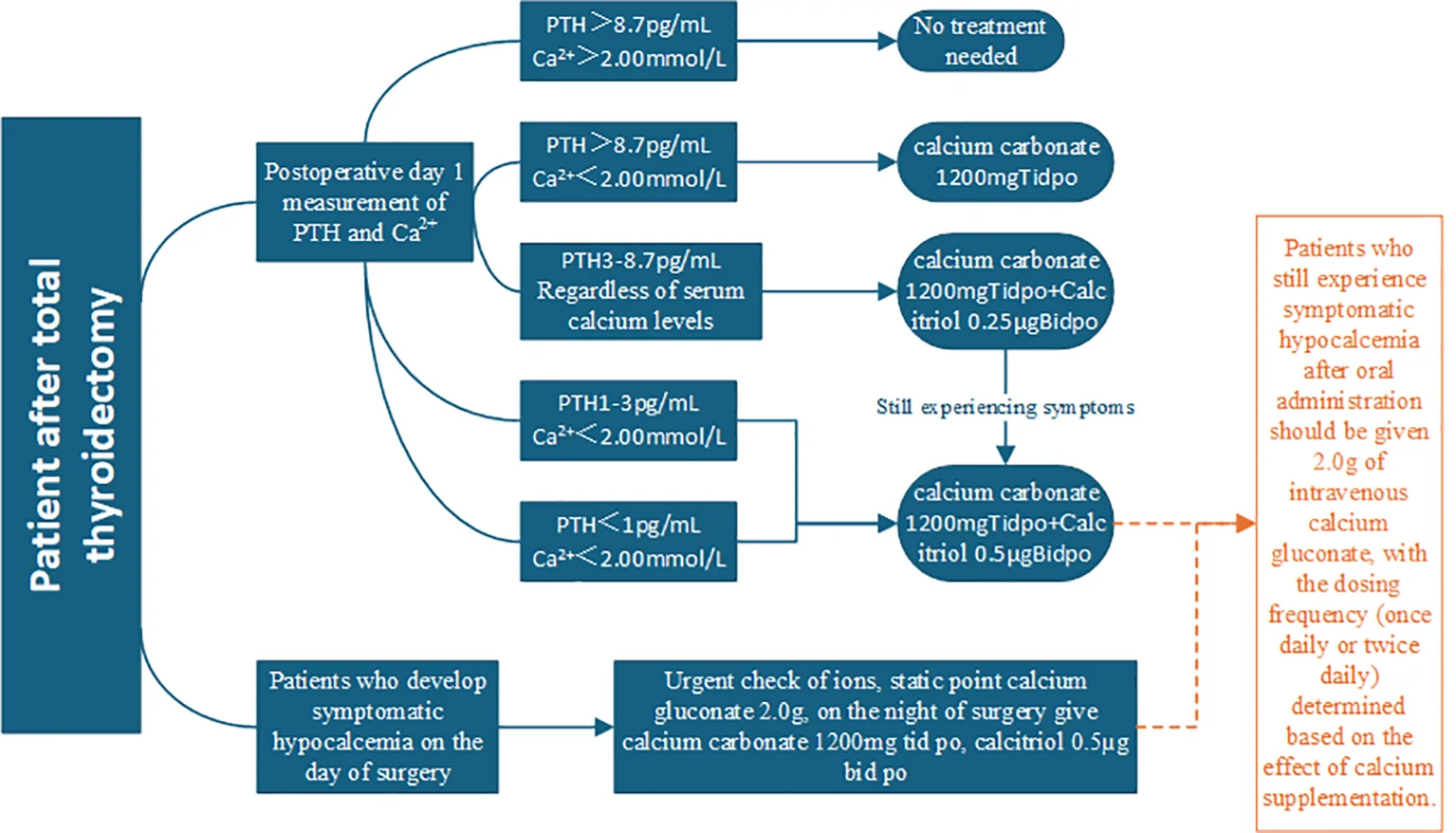
Flow chart for calcium supplementation in hypocalcemia.

For all three groups, serum PTH and calcium concentrations were measured from the cephalic vein at the elbow of the non-transplanted brachioradialis at the same time points. The BAT group additionally underwent measurement from the transplanted side. All patients experiencing HypoPT events received calcium supplementation according to the hypocalcemia management protocol (**Figure 5**).

### 2.4 Research design

#### 2.4.1 Experiment 1

Comparative analysis of serum PTH concentrations from bilateral elbow cephalic veins was performed to establish the survival and viability criteria for brachioradialis parathyroid autotransplantation, with evaluation of the safety, efficacy, and monitorability of this technique.

#### 2.4.2 Experiment 2

For all three groups of patients undergoing TT with bilateral CND, comparative analysis of serum PTH and calcium levels was conducted to determine the efficacy of brachioradialis parathyroid autotransplantation in reducing postoperative HypoPT, as well as to validate the accuracy of the proposed graft survival criteria.

### 2.5 Relevant criterions and definitions

#### 2.5.1 HypoPT

Permanent HypoPT: Serum PTH not normalized within 6 months, or calcium cannot be maintained normally despite PTH reaching the lower limit. Other cases were defined as transient HypoPT (9).

#### 2.5.2 Hypocalcemia

Albumin-corrected total serum calcium below 8.4 mg/dL (2.1 mmol/L). The presence of tetany, muscle cramps, or perioral numbness was considered indicative of symptomatic hypocalcemia (10).

#### 2.5.3 Graft survival

Previous studies used a transplanted-to-non-transplanted arm PTH concentration ratio ≥ 1.5 as the criterion for graft survival (11). In the present investigation, a ratio ≥ 1.2 was adopted as the viability criterion for parathyroid autografts.

### 2.6 Sample size calculation and statistical method

Sample size calculation was performed. Based on previously published data (13% in the graft group vs. 36% in the control group) (12), a sample size of 54 participants per group was calculated to detect an expected 23% between-group difference in the incidence of postoperative HypoPT, with a statistical power of 80% and a significance level of 5%. To account for a potential 10% loss to follow-up, we enrolled 60 patients in each group. To minimize selection bias caused by non-random group assignment driven by patient preference between BAT and SAT groups, propensity score matching (PSM) was performed for both the BAT versus SAT and BAT versus ISP comparisons with 1:1 nearest-neighbor matching at a caliper threshold of 0.05. The matching covariates included age, sex, preoperative serum calcium, preoperative PTH, and preoperative 25-hydroxyvitamin D3, all of which are potential confounders associated with postoperative PTH levels. Both the original cohort and the PSM-matched cohort were analyzed, which were used to demonstrate the robustness of our findings against selection bias. Statistical analysis was performed using IBM SPSS Statistics for Windows (version 27.0; IBM Corporation, Armonk, NY, USA). Normality testing preceded all analyses of continuous variables. Normally distributed data were compared between two groups using the independent samples *t*-test and among three groups using analysis of variance, and expressed as the mean ± standard deviation. Non-normally distributed data were compared between two groups using the Mann–Whitney U test and among three groups using the Kruskal–Wallis H test, and are presented as the median with interquartile range (P_25_, P_75_). Categorical variables were analyzed using the chi-square test or Fisher’s exact test. Logistic regression was employed to evaluate the relationship between PTG weight and graft survival. Receiver operating characteristic (ROC) curve analysis with calculation of the area under the curve (AUC) was conducted to assess the predictive capability of gland weight, including determination of the optimal cutoff point, sensitivity, and specificity. A two-tailed *p* value < 0.05 was considered statistically significant.

## 3 Results

### 3.1 Experiment 1

#### 3.1.1 Characteristics of PTH gradient in the bilateral arms of control subjects

To establish a scientific criterion to assess parathyroid graft survival following brachioradialis transplantation, 30 subjects with no history of thyroid surgery were enrolled as controls. The bilateral brachioradialis were designated as Arm 1 or Arm 2, and the bilateral brachioradialis PTH levels were compared (baseline characteristics are presented in **Table 1**). The results demonstrated that the PTH concentration ratio between the bilateral brachioradialis did not exceed 1.2-fold in any subject (**Supplementary Table 1**), with a maximum ratio of 1.16, minimum of 1.00, and median of 1.03. Based on this population distribution, we propose that a serum PTH concentration ratio ≥1.2 between the transplanted and non-transplanted brachioradialis may serve as a potential biological indicator to evaluate viability of the brachioradialis parathyroid graft.

**Table 1:** Baseline characteristics of healthy controls.

Variables	n/mean ± SD	Percent (%)
Sex		
Male	6	20.00
Female	24	80.00
Age, years	50.00 ± 14.67	
BMI, kg/m^2^	24.87 ± 3.10	
Preoperative hemoglobin, g/L	140.60 ± 15.52	
Preoperative albumin, g/L	44.60 (42.83, 47.35)	
Diabetes	5	16.67
Hypertension	10	33.33
Preoperative calcium, mmol/L	2.28 ± 0.10	
Preoperative PTH, pg/mL	49.20 (41.88, 59.10)	
Preoperative Tg, ng/mL	19.23 (89.85, 224.50)	
Preoperative TgAb, IU/mL	0.87 (0.00, 12.63)	

BMI, body mass index.

#### 3.1.2 Survival analysis of different criteria after parathyroid brachioradialis transplantation

The cohort of Experiment 1 consisted of 100 patients (24 males and 76 females; mean age, 45.83 ± 10.33 years) undergoing parathyroid autotransplantation, which included 10 patients with benign thyroid tumors and 90 with malignant thyroid tumors. Follow-up time points were designated as POD 1, POD 3, POW 1, POW 2, POM 1, and POM 3 for short-term assessment, whereas long-term follow-up was defined at POM 6.

##### 3.1.2.1 Survival according to different criteria

When employing a bilateral PTH concentration ratio ≥1.5 as the viability criterion, the short-term survival rate of transplanted PTGs exhibited a progressive upward trend over time (**Table 2**), with a long-term survival rate of 74% at POM 6. Baseline characteristic analysis (**Supplementary Table 2**) revealed no statistically significant differences between the survival group (n = 74) and non-survival group (n = 26) in terms of sex, age, BMI, preoperative laboratory parameters (hemoglobin, albumin, serum calcium, PTH, Tg, and TgAb levels), comorbidities (diabetes mellitus and hypertension), tumor-related clinical features (tumor location, size, multifocality, extrathyroidal extension, number of lymph nodes dissected, and tumor stage), or PTG weight (all, *p* > 0.05), thereby confirming comparability between the two cohorts. In contrast, utilizing our proposed criterion of a bilateral PTH concentration ratio ≥1.2 increased the long-term survival rate to 83% at POM 6, with short-term survival rates also demonstrating a time-dependent ascending pattern (**Table 2**). Baseline analysis (**Table 3**) demonstrated that, unlike the findings with the 1.5-fold criterion, PTG weight was significantly lower in the non-survival group than the survival group (32.00 [23.00, 56.00] mg vs. 46.00 [34.00, 60.00] mg, *p* = 0.046), whereas there were no statistically significant differences in all other baseline parameters between the two groups (*p* > 0.05).

**Table 2:** Comparison of parathyroid graft survival rates at different follow-up time points under different survival criteria.

Time	At 1.5-fold ratio ^#^		At 1.2-fold ratio ^#^		*p* ^*^
	Number of survivors (n)	Graft survival rate (%)	Number of survivors (n)	Graft survival rate (%)	
POD 1	0	0%	11	11%	< 0.001
POD 3	0	0%	24	24%	< 0.001
POW 1	30	30%	52	52%	0.002
POW 2	48	48%	63	63%	0.033
POM 1	56	56%	70	70%	0.040
POM 3	62	62%	70	70%	0.232
POM 6	74	74%	83	83%	0.121

^*^ PTH concentration ratio between the transplanted and non-transplanted sides.

**Table 3:** Patient baseline profiles under the 1.2-fold criterion.

Variables	Non-surviving group (n = 17)		Survival group (n = 83)		*p*
	n/mean ± SD/P_50_ (P_25_, P_75_)	Percent (%)	n/mean ± SD/P_50_ (P_25_, P_75_)	Percent (%)	
Sex					0.547
Male	5	29.41	19	22.89	
Female	12	70.59	64	77.11	
Age, years	42.65 ± 9.44		46.48 ± 10.43		0.164
BMI, kg/m^2^	26.42 ± 4.21		24.76 ± 3.83		0.111
Preoperative hemoglobin, g/L	140.65 ± 21.36		139.95 ± 13.54		0.899
Preoperative albumin, g/L	45.97 ± 1.97		45.76 ± 2.89		0.725
Diabetes	2	11.76	11	13.25	1.000
Hypertension	4	23.53	20	24.10	1.000
Preoperative calcium, mmol/L	2.32 ± 0.09		2.37 ± 0.11		0.115
Preoperative PTH, pg/mL	44.70 (32.90, 58.40)		44.70 (34.55, 54.85)		0.896
Preoperative Tg, ng/mL	13.94 (7.08, 23.26)		9.81 (4.25, 29.54)		0.328
Preoperative TgAb, IU/mL	1.38 (0.90, 2.70)		1.03 (0.58, 19.22)		0.872
Tumor location ^#^					0.507
Unilateral	14	87.50	57	77.00	
Bilateral	2	12.50	17	23.00	
Tumor size, mm^#^	9.00 (3.00, 20.00)		6.00 (4.00, 10.00)		0.581
Multifocality^#^					0.768
Single	12	75.00	51	68.92	
Multiple (≥2)	4	25.00	23	31.08	
Extrathyroid invasion^#^					0.295
Yes	9	56.25	31	41.89	
No	7	43.75	43	58.11	
Lymph node metastases^#^					0.183
Yes	8	50.00	24	32.43	
No	8	50.00	50	67.57	
Number of LND^#^	8.50 (3.50, 11.50)		6.00 (3.00, 8.00)		0.172
Tumor staging ^†,#^					0.326
Phase 1	15	93.80	73	98.60	
Phase 2	1	6.30	1	1.40	
PTG weight, mg	32.00 (24.00, 54.00)		46.00 (34.50, 59.00)		0.046

^#^ Total of 90 patients with tumors. BMI, body mass index.

^†^ American Joint Committee on Cancer – Thyroid Cancer TNM Staging System (8th ed, 2017). The bold values indicate whether the tumor location involves a single glandular lobe or bilateral glandular lobes.

##### 3.1.2.2 Comparison of the results of the two judgments

Comparative analysis revealed that survival rates were superior under the 1.2-fold criterion as compared to the 1.5-fold criterion at each short-term follow-up time point, reaching statistical significance at POD 1, POD 3, POW 1, POW 2, and POM 1 (all, *p* < 0.05). Conversely, no statistically significant differences were observed at POM 3 and POM 6 (both, *p* > 0.05) (**Table 2**).

#### 3.1.3 Predictive value and time-dependent analysis of PTG weight under the 1.2-fold survival criterion for graft survival

##### 3.1.3.1 Prediction efficiency of the ROC curve

**Figure 6:**
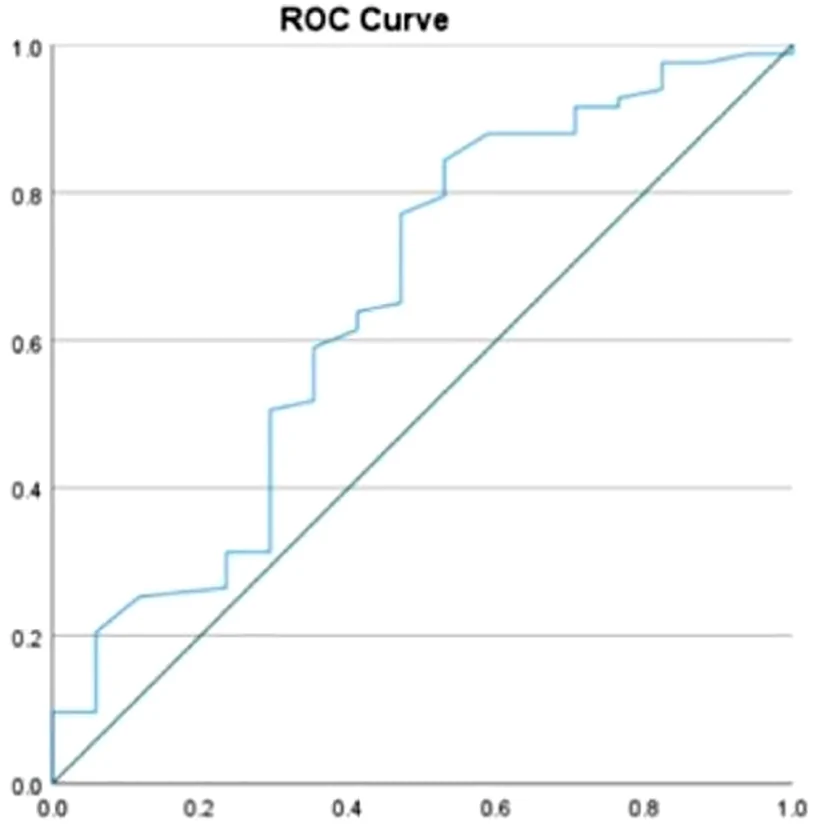
ROC curve analysis of PTG weight under the 1.2-fold criterion.

ROC curve analysis was conducted to evaluate the predictive value of PTG weight for graft survival. The results (**Figure 6**; **Table 4**) revealed an AUC of 0.654 (95% confidence interval [CI]: 0.532–0.776), indicating moderate predictive efficacy. The optimal cutoff value was 28.5 mg, yielding sensitivity of 80.8%, specificity of 50.0%, and Youden’s index of 0.314.

**Table 4:** Youden index of parathyroid weight ROC analysis under the 1.2-fold criterion.

Variable	AUC	Best cutoff	Sensitivity (%)	Specificity (%)	Maximum Youden index^*^
Parathyroid weight, mg	0.654	28.5	80.8	50.0	0.314

AUC, area under the curve; *Sensitivity + specificity – 1.

##### 3.1.3.2 Association of parathyroid weight with graft survival at different time points

Longitudinal analysis demonstrated a time-dependent association between PTG weight and graft survival status (**Table 5**). At POD 1, POD 3, and POM 6, PTG weight was significantly greater in the survival group than the non-survival group (all, *p* < 0.05), whereas there were no statistically significant intergroup differences at POW 1, POW 2, and POM 3 (all, *p* > 0.05).

**Table 5:** Temporal correlation between transplanted PTG weight and survival rate.

Time	PTG Weight (mg)^*^				*p*
	Surviving graft [n/P_50_ (P_25_, P_75_)]		Non-surviving graft [n/P_50_ (P_25_, P_75_)]		
POD 1	11	68.00 (45.00, 74.00)	89	41.00 (30.50, 54.50)	0.003
POD 3	24	52.00 (38.25, 60.00)	76	41.00 (29.25, 55.75)	0.038
POW 1	52	49.50 (35.00, 59.50)	48	39.00 (28.00, 57.00)	0.052
POW 2	63	47.00 (32.00, 60.00)	37	40.00 (30.00, 53.00)	0.286
POM 1	70	47.00 (34.00, 60.00)	30	40.50 (26.50, 55.75)	0.113
POM 3	70	42.50 (34.00, 57.75)	30	44.50 (27.75, 58.00)	0.635
POM 6	83	46.00 (34.00, 60.00)	17	32.00 (23.00, 56.00)	0.046

^*^ Each PTG was individually weighed before transplantation.

### 3.2 Experiment 2

#### 3.2.1 Comparison of the clinical effect of PTG preservation *in situ* and two transplantation sites

The cohort of Experiment 2 consisted of 180 patients undergoing TT combined with bilateral CND, who were allocated into three groups (n = 60 each): ISP, BAT, and SAT. All patients were followed-up for 6 months postoperatively. There were no significant differences in baseline characteristic among the three groups in regard to sex, age, BMI, preoperative serum calcium, preoperative PTH, preoperative 25-OH-D3, tumor-related clinical features (location, size, multifocality, number of lymph nodes dissected, tumor stage), or number of PTGs identified (all, *p* > 0.05) (**Table 6**). To further address potential confounding effects from baseline characteristics, PSM was additionally performed. After matching, 50 patients per group were retained, and the baseline characteristics remained well balanced among the three groups (all, *p* > 0.05) (**Supplementary Table 3**).

**Table 6:** Comparison of clinicopathological characteristics among the three patient groups.

Variables	ISP (n = 60)		BAT (n = 60)		SAT (n = 60)		*p*
	n/mean ± SD/P_50_ (P_25_, P_75_)	Percent (%)	n/mean ± SD/P_50_ (P_25_, P_75_)	Percent (%)	n/mean ± SD/P_50_ (P_25_, P_75_)	Percent (%)	
Sex							0.521
Male	11	18.30	16	26.70	15	25.00	
Female	49	81.70	44	73.30	45	75.00	
Age, years	48.90 ± 9.05		47.07 ± 10.21		50.02 ± 9.49		0.238
BMI, kg/m^2^	25.45 ± 3.52		25.18 ± 4.28		26.64 ± 3.16		0.071
Preoperative calcium, mmol/L	2.37 ± 0.11		2.35 ± 0.10		2.35 ± 0.11		0.565
Preoperative PTH, pg/mL	45.20 (35.50,59.25)		45.10 (30.40,63.35)		43.80 (34.50,64.50)		0.979
Preoperative 25-OH-D_3_, ng/mL	12.90 (9.40,16.95)		13.70 (9.85,15.90)		15.20 (11.00,17.05)		0.090
Tumor location ^#^							
Unilateral	19	38.78	32	56.14	29	59.18	
Bilateral	30	61.22	25	43.86	20	40.82	
Tumor size, mm ^#^	8.00 (5.00,12.00)		8.00 (6.00,12.00)		8.00 (5.00,14.00)		0.613
Multifocality ^#^							0.184
Single	15	30.61	26	45.61	23	46.94	
Multiple (≥2)	34	69.39	31	54.39	26	53.06	
Number of LND^#^	8.00 (4.00,10.00)		7.00 (3.00,10.00)		5.00 (2.00,10.00)		0.583
Tumor staging ^†,#^							0.135
Phase 1	43	87.76	55	96.50	47	95.90	
Phase 2	6	12.24	2	3.50	2	4.10	
PTG yield	3.00 (2.00,3.00)		3.00 (2.50,3.00)		3 (3.00,3.00)		0.367

^#^ Total of 155 patients with tumors.

BMI, body mass index.

^†^ American Joint Committee on Cancer – Thyroid Cancer TNM Staging System (8th ed, 2017). The bold values indicate whether the tumor location involves a single glandular lobe or bilateral glandular lobes.

##### 3.2.1.1 Postoperative HypoPT and serum calcium levels

The incidence rates of postoperative HypoPT across the three groups are presented in **Table 7**. On POD 1, the rates for the ISP, BAT, and SAT groups were 61.67% (37/60), 35.00% (21/60), and 55.00% (33/60), respectively, with statistically significant intergroup differences (*p* = 0.010). Pairwise comparisons revealed that the incidence of HypoPT was significantly lower in the BAT group than the ISP and SAT groups (both, *p* < 0.05). At POD 3, the incidence declined in all groups (ISP, 51.67%; BAT, 26.67%; SAT, 48.33%) and intergroup differences remained significant (*p* = 0.011), with the BAT group showing a significantly lower rate than both the ISP and SAT groups (both, *p* < 0.05). At POW 1, the BAT group maintained a significantly lower incidence (23.33%) compared with the ISP group (41.67%) (*p* < 0.05). From POW 1 onward, no significant intergroup differences were observed (all, *p* > 0.05). By POM 6, the incidence had decreased to 6.67% in the ISP group and 5.00% in both the BAT and SAT groups. The PSM analysis yielded consistent results (**Supplementary Table 4**). On POD 1, the incidence rates were 62.00% (ISP), 30.00% (BAT), and 58.00% (SAT), with significant intergroup differences (*p* = 0.002); the BAT group was significantly lower than both the ISP and SAT groups (both, *p* < 0.05). At POD 3, the rates were 50.00% (ISP), 22.00% (BAT), and 50.00% (SAT), with the BAT group again significantly lower than the other two groups (both, *p* < 0.05). At POW 1, the BAT group maintained a significantly lower incidence (18.00%) compared with the ISP (42.00%) and SAT (42.00%) groups (both, *p* < 0.05). No significant intergroup differences were observed from POW 2 to POM 6 (all, *p* > 0.05). By POM 6, the incidence had decreased to 6.00% in the ISP and BAT groups and 4.00% in the SAT group. Postoperative serum calcium levels at all measured time points showed no significant intergroup differences in either the original or PSM analysis (all, *p* > 0.05) (**Supplementary Tables 5, S6**).

**Table 7:** Postoperative HypoPT occurrence among the three patient groups (n = 60).

Time	ISP (n/%)	BAT (n/%)	SAT (n/%)	*p*
POD 1	37/61.67	21/35.00 ^a^	33/55.00 ^b^	0.010
POD 3	31/51.67	16/26.67 ^a^	29/48.33 ^b^	0.011
POW 1	25/41.67	14/23.33 ^a^	23/38.33	0.079
POW 2	10/16.67	7/11.67	13/21.67	0.340
POM 1	8/13.33	5/8.33	6/10.00	0.662
POM 3	5/8.33	3/5.00	3/5.00	0.679
POM 6	4/6.67	3/5.00	3/5.00	0.900

^a^ *p* < 0.05 vs. ISP group.

^b^ *p* < 0.05 vs. BAT group.

##### 3.2.1.2 Postoperative survival of BAT patients under different survival criteria

**Figure 7:**
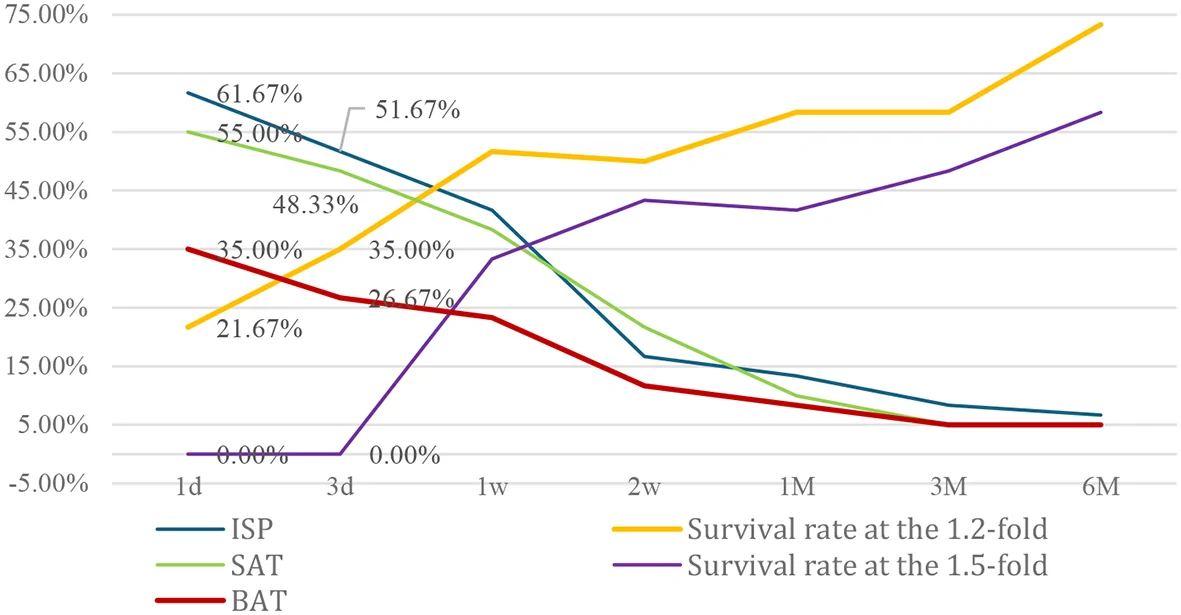
Postoperative HypoPT incidence of the three groups and survival rates under the different criteria. Data are presented as the survival rate (%). The purple curve denotes the 1.5-fold criterion and the orange curve denotes the 1.2-fold criterion. Curves for the three patient groups are defined as follows: ISP (blue), BAT (red), and SAT (green). The abscissa indicates the follow-up time and the ordinate indicates the survival rate.

Dynamic trend and comparative analyses of graft survival rates, as determined by the 1.5-fold criterion (purple curve) and 1.2-fold criterion (orange curve), revealed (**Figure 7**; **Table 8**): Under the 1.2-fold survival criterion, BAT patients exhibited significantly higher graft survival rates at POD 1, POD 3, and POW 1 as compared to the 1.5-fold standard (21.67%, 35.00%, 51.67% vs. 0.00%, 0.00%, 33.33%, respectively; all *p* < 0.05). No statistically significant differences in survival rates were observed between the two criteria at POW 2, POM 1, POM 3, and POM 6 (all, *p* > 0.05). The PSM analysis yielded consistent results (**Supplementary Figure 1**; **Supplementary Table 7**). Under the 1.2-fold survival criterion, BAT patients exhibited significantly higher graft survival rates at POD 1, POD 3, and POW 1 as compared to the 1.5-fold standard (26.00%, 34.00%, 48.00% vs. 0.00%, 0.00%, 28.00%, respectively; all *p* < 0.05). No statistically significant differences in survival rates were observed between the two criteria at POW 2, POM 1, POM 3, and POM 6 (all, *p* > 0.05).

**Table 8:** BAT survival rates at different follow-up time points by survival criteria.

Time	At 1.5-fold ratio^*^		At 1.2-fold ratio ^*^		*p*
	Number of survivors (n)	Graft survival rate (%)	Number of survivors (n)	Graft survival rate (%)	
POD 1	0	0.00%	13	21.67%	< 0.001
POD 3	0	0.00%	21	35.00%	< 0.001
POW 1	20	33.33%	31	51.67%	0.042
POW 2	26	43.33%	30	50.00%	0.464
POM 1	25	41.67%	35	58.33%	0.068
POM 3	29	48.33%	35	58.33%	0.272
POM 6	34	58.33%	44	73.33%	0.083

*PTH concentration ratio between the transplanted and the non-transplanted sides.

## 4 Discussion

The number of PTGs varies anatomically, with cadaveric studies reporting 2–11 glands per individual. While most individuals possess four glands, 4.9%–10.7% harbor supernumerary glands (predominantly five), 2.0%–9.7% have three glands, and approximately 1.8%–4.1% possess only two glands (13–15). Based on their spatial relationship with the thyroid gland, PTGs are classified as Type A (adherent to the thyroid surface, partially/fully embedded within the capsule, or intrathyroidal), which are more challenging to preserve *in situ*, and Type B (perithyroidal, intrathymic, or thymic/mediastinal blood supply subtype), which are more readily identified and preserved (16). Previous investigations have demonstrated that the incidences of transient and permanent HypoPT following TT (TT) alone are 27.7% and 6.3%, respectively, which increase to 36.1% and 7.0% with concomitant unilateral CND, and further escalate to 51.9% and 16.2% with bilateral CND (17). Contemporary surgical practice incorporates several parathyroid preservation strategies, including meticulous capsular dissection to protect vascular integrity (4), utilization of tracers, such as carbon nanoparticles or mitoxantrone hydrochloride, to enhance parathyroid identification through negative staining (5, 18), and autotransplantation of devascularized or inadvertently resected glands (7). Nevertheless, transient and permanent HypoPT remain significant complications following thyroid surgery.

In 1926, Lahey (19) first demonstrated that parathyroid autotransplantation could prevent HypoPT. Sun et al. (20) reported that in 358 TT cases, the incidence of permanent HypoPT decreased progressively with an increasing number of autotransplanted glands (no transplant, 4.4%; one gland, 1.4%; two glands, 0.7%; three glands, and 0.0%), reaffirming the clinical value of this technique. Although parathyroid autotransplantation has been widely adopted, controversy persists regarding objective assessment of graft viability. The sternocleidomastoid has served as the established transplantation site, attributed to its robust blood flow and high oxygen tension that may enhance graft survival (21). As the preferred clinical location, the technical advantage of the sternocleidomastoid lies in obviating the need for additional skin incisions. However, a critical limitation is the inability to verify graft viability. To address this restriction, Wells et al. (22) first introduced brachioradialis autotransplantation in 1975. Subsequently, Cavallaro et al. (23) reported a 3-month graft survival rate of 96% among 25 brachioradialis autotransplantations in 348 thyroidectomy patients, thereby establishing the safety and efficacy of this approach. Li et al. (24) further highlighted that brachioradialis transplantation is technically straightforward, easily implemented, and enables real-time functional assessment.

This study innovatively proposes that a PTH concentration ratio ≥ 1.2 between the graft side and the non-graft side serves as the diagnostic criterion for graft survival. This finding challenges the conventional 1.5-fold criterion widely adopted in previous literature (11). The results of Experiment 1 in our study showed that the maximum PTH concentration ratio of the bilateral forearms in the control population was only 1.16, with a median of 1.03. This indicates that there is an inherent slight physiological gradient in the bilateral upper extremities of the human body, with a magnitude lower than 1.2-fold. Therefore, requiring graft function to exceed that of the healthy side by 50% (the 1.5-fold criterion) is inconsistent with human physiological characteristics. Further analysis reveals that the application of the 1.5-fold criterion would erroneously evaluate the early postoperative (Day 1/Day 3) graft survival rate as 0%, leading to misdiagnosis of “pseudo-graft inactivation”. In contrast, the 1.2-fold criterion is more in line with the anatomical and physiological basis of the human body, enabling accurate identification of the graft’s stunning phase before vascularization and maturation, as well as its subsequent functional reconstruction process. The proposed criterion not only avoids overly pessimistic assessment of early graft function but also provides a more scientific basis for clinicians to implement early postoperative management strategies, such as calcium supplementation regimens.

After establishing the diagnostic criteria, we further explored the biological factors influencing graft survival. ROC curve analysis identified parathyroid gland weight as a supplementary auxiliary factor for predicting long-term graft survival, with an optimal cutoff value of 28.5 mg. The AUC of 0.654 indicates modest discriminatory performance, suggesting that parathyroid gland weight alone has limited utility as a standalone clinical predictor. However, this quantitative parameter retains practical operational value as an intraoperative morphological reference. Specifically, the high sensitivity (80.8%) associated with this cutoff suggests that maintaining a graft weight above this threshold significantly improves the likelihood of survival, providing surgeons with a tangible quantitative standard to avoid excessive subdivision of tiny fragments. The pathophysiological mechanism underlying this finding can be plausibly explained as follows: smaller tissue fragments (< 28.5 mg) may encounter a dual adverse dilemma. On one hand, although small-sized tissues theoretically facilitate nutrient diffusion, the total number of parathyroid cells may be insufficient to maintain the minimal physiological function during the revascularization window of ectopic transplantation. On the other hand, an overly small tissue volume leads to a larger specific surface area, which exacerbates oxidative stress injury in the early post-transplantation stage. These findings suggest that surgeons should not blindly adopt multi-site microfragment implantation. Instead, guided by the 28.5 mg cutoff identified in this study, an adequate tissue volume should be guaranteed per single graft site to improve ischemic tolerance. We propose that this value serves as a “safety threshold” to ensure sufficient cell mass during the critical revascularization period. In addition, although high local oxygen partial pressure represents an ideal transplantation condition, and elevated hemoglobin concentration theoretically enhances oxygen-carrying capacity and tissue oxygenation (25), our study found no association between preoperative hemoglobin levels and long-term graft survival, consistent with the findings of Li et al. (24). Calcium levels are known to regulate parathyroid hormone secretion through negative feedback (26–29). As all patients in our cohort received standardized calcium supplementation postoperatively to mitigate hypocalcemic symptoms and adverse effects, we did not analyze the correlation between postoperative serum calcium and graft survival.

The long-term survival rate in Experiment 1 was 83% (83/100), comparable to 85.7% reported by Li et al. (24) but lower than 92.8% reported by Cavallaro et al. (30). This discrepancy prompted us to conduct Experiment 2 to validate the efficacy of brachioradialis transplantation to reduce the incidence of postoperative HypoPT. Our findings revealed permanent HypoPT rates of 6.67% (4/60), 5.00% (3/60), and 5.00% (3/60) for the ISP, BAT, and SAT groups, respectively, with no statistically significant differences (*p* > 0.05). Thus, irrespective of the parathyroid preservation technique employed, the incidence of permanent HypoPT remained unchanged after TT with bilateral CND, underscoring the critical importance of preoperative patient counseling regarding this complication to mitigate physician-patient disputes.

Notably, the BAT group exhibited a markedly lower incidence of early HypoPT at POD 1 (35% [21/60]) and POD 3 (26.67% [16/60]) relative to the ISP group (61.67% [37/60] and 51.67% [31/60]) and SAT group (55.00% [33/60] and 48.33% [29/60]; *p* < 0.05). This represents reductions of 26.67% (16/60) and 25.00% (15/60) at POD 1 and POD 3, respectively, relative to the ISP group. These findings suggest that parathyroid glands left *in situ* during TT+CND may undergo a process of devitalization despite anatomical preservation. During total thyroidectomy combined with bilateral central neck dissection, the vascular pedicles that sustain the parathyroid glands, such as branches of the inferior thyroid artery, often have to be sacrificed to achieve radical tumor resection. Although the glands appear morphologically intact *in situ* on gross inspection, their blood supply is disrupted, resulting in devitalization and eventual loss of function. In contrast, forearm brachioradialis muscle transplantation, though regarded as a salvage procedure, allows the transplanted parathyroid glands to re-establish blood perfusion relying on the abundant vascular network of the forearm muscle. This approach avoids persistent ischemic necrosis caused by vascular pedicle injury when the parathyroid glands are preserved *in situ*. We further analyzed the impact of different viability criteria on the incidence of early HypoPT. Under the 1.5-fold criterion, no grafts were deemed viable at POD 1 and POD 3. However, applying the 1.2-fold criterion revealed graft survival rates of 21.67% (13/60) and 35% (21/60) at POD 1 and POD 3, respectively. Given that graft survival rates under both criteria increased progressively over time, our results confirm that early graft viability at POD 1 and POD 3, as assessed by the 1.2-fold standard, accounts for the reduced incidence of early HypoPT in the BAT group. We therefore propose that a PTH concentration ratio ≥1.2 between the transplant and contralateral arms represents a more appropriate and precise viability criterion for brachioradialis parathyroid autotransplantation. In addition, During the 6-month follow-up period in Experiment 1, transient inactivation followed by reactivation was observed in 32 patients. Among the 17 patients with non-viable grafts, 2 showed no viability indicators at any time point, 4 became nonfunctional by POW 1, 5 by POM 1, 4 by POM 3, and 2 by POM 6. The underlying mechanisms for these phenomena remain under investigation.

This study is the first to investigate the predictive value of PTG weight for long-term graft survival in brachioradialis autotransplantation and to establish the clinical utility of the 1.2-fold PTH ratio as a more sensitive viability criterion. While the present study demonstrated novel findings, it still has several limitations that should be acknowledged. First, the allocation in Experiment 2 was not randomized but was based on patient preference, which inherently carries a risk of selection bias. Although we attempted to mitigate this by performing PSM to generate a balanced cohort of 50 patients per group, residual confounding factors may still exist. The non-randomized design remains a constraint on the strength of the comparative conclusions between the BAT and SAT groups. Second, our 6-month follow-up period is insufficient, as graft function may exhibit long-term fluctuations. Future studies require cohorts with ≥ 2–3 years follow-up periods to clarify the mechanisms and clinical significance of “transient inactivation with subsequent reactivation”. Third, graft weight’s moderate AUC (0.654) indicates limited standalone prediction. Graft survival depends on multifactorial dynamics (microvascular revascularization, oxidative stress), not merely static morphology. Thus, graft weight should serve as a supplementary morphological reference, not a definitive prognostic biomarker. Future models integrating functional markers (e.g., PTH, microvascular imaging) are needed. Fourth, the proposed 1.2-fold PTH ratio, while physiologically rational and validated internally, lacks external validation. As this threshold was derived from a limited control cohort, it should be considered hypothesis-generating. Multi-center, prospective studies with larger sample sizes are required to confirm its generalizability. Fifth, despite various protective measures, the incidence of permanent HypoPT remains as high as 5% after bilateral CND, necessitating exploration of novel interventions, such as parathyroid stem cell transplantation, organoid allotransplantation, sustained-release PTH biocompatible scaffolds, or gene therapy. In addition, differences in the immune microenvironment and vascularization kinetics between the brachioradialis and sternocleidomastoid transplantation sites remain uncharacterized; future investigations could employ single-cell sequencing and spatial transcriptomics to dissect these molecular mechanisms. Finally, comprehensive assessment systems integrating PTH, serum calcium, vitamin D, and bone metabolism markers should be established, and the impact of individualized calcium supplementation protocols on graft function further explored. With advances in precision surgery and regenerative medicine, parathyroid transplantation will evolve from an “empirical procedure” to an intelligent “prediction-decision-verification” paradigm, ultimately enabling individualized and precise management of postoperative parathyroid function.

## 5 Conclusion

In summary, parathyroid autotransplantation into the brachioradialis of the forearm is safe and feasible. A 1.2-fold PTH ratio between the transplanted and non-transplanted arms serves as a more sensitive and reliable criterion for graft survival. This transplantation strategy significantly reduces the incidence of early hypoparathyroidism after total thyroidectomy with bilateral central neck dissection, although none of the three strategies can mitigate the risk of permanent hypoparathyroidism. Therefore, brachioradialis autotransplantation is recommended for parathyroid glands with poor intraoperative blood supply or those inadvertently excised.

## Data Availability

Data contain sensitive patient information; public deposition is not applicable. Requests to access the datasets should be directed to CS 457614014@qq.com.
