# Supplementary material for: Parathyroid autotransplantation into the brachioradialis protects parathyroid function during total thyroidectomy
Source: Front Endocrinol (Lausanne). 2026 Aug 18;17:1852443. doi: 10.3389/fendo.2026.1852443 (PMC13529502; doi:10.3389/fendo.2026.1852443)
Supplement: Supplementary file 1 [file DataSheet1.pdf]

TABLE S1 Bilateral PTH Levels in the Normal Population

No.	PTH concentration (pg/ml)		Ratio*
	Arm 1	Arm 2	
1	33.0	32.5	1.02
2	102.0	93.6	1.09
3	53.9	46.3	1.16
4	49.7	48.2	1.03
5	30.7	29.8	1.03
6	64.4	61.8	1.04
7	58.4	58.2	1.00
8	48.2	42.0	1.15
9	85.9	83.8	1.03
10	87.4	80.0	1.09
11	49.4	47.7	1.04
12	53.2	49.9	1.07
13	51.7	50.1	1.03
14	51.7	50.1	1.03
15	48.4	47.4	1.02
16	39.1	38.2	1.02
17	49.5	48.0	1.03
18	54.1	50.6	1.07
19	42.3	41.5	1.02
20	30.1	30.0	1.00
21	77.4	74.9	1.03
22	55.9	49.8	1.12
23	54.9	48.6	1.13
24	59.4	58.2	1.02
25	83	80	1.04
26	52.7	50.1	1.05
27	48.4	47.2	1.03
28	39.1	37.8	1.03
29	42.7	41.5	1.03
30	64.4	62.3	1.03

*, bilateral forearm PTH concentration ratio.

TABLE S2 Patient Baseline Profiles Under the 1.5-Fold Survival Standard

Variables	Non-surviving group (n = 26)		Survival Group (n = 74)		*P* value
	n/mean ± SD	Percent (%)	n/mean ± SD	Percent (%)	
Gender					0.685
Male	7	26.90	17	23.00	
Female	19	73.10	57	77.00	
Age, yr	45.35 ± 10.04		46.00 ± 10.49		0.783
BMI, kg/m2	25.99 ± 4.36		24.71 ± 3.73		0.155
Preoperative hemoglobin, g/L	138.04 ± 19.67		140.78 ± 13.10		0.513
Preoperative albumin, g/L	45.65 ± 2.30		45.85 ± 2.90		0.761
Diabetes	6	46.15	7	53.85	0.094
Hypertension	7	29.17	17	70.83	0.685
Preoperative calcium, mmol/L	2.33 ± 0.10		2.37 ± 0.11		0.109
Preoperative PTH, pg/ml	44.10(32.90,59.40)		45.70(34.30,55.20)		0.832
Preoperative Tg, ng/ml	14.08(6.73,23.26)		9.77(4.42,29.81)		0.427
Preoperative TgAb, IU/ml	1.40(0.72,3.10)		1.02(0.59,20.20)		0.975
Tumor location #					0.677
Unilateral	19	76.00	52	80.00	
Bilateral	6	24.00	13	20.00	
Tumor size, mm #	8.00(3.00,12.00)		6.00(4.00,10.00)		0.721
Multifocality #					0.441
Single	16	64.00	47	72.30	
Multiple (≥ 2)	9	36.00	18	27.70	
Extrathyroid invasion #					0.371
Yes	13	52.00	27	41.50	
No	12	48.00	38	58.50	
Lymph node metastases #					0.126
Yes	12	48.00	20	30.80	
No	13	52.00	45	69.20	
Number of LND #	7.00(4.00,10.00)		6.00(3.00,8.00)		0.280
Tumor Staging †,#					0.075
Phase 1	23	92.00	65	100.00	
Phase 2	2	8.00	0	0.00	
Parathyroid gland weight, mg	43.50(27.00,58.00)		43.50(34.00,57.00)		0.703

#, Number of patients with tumor was 90 in the study. BMI, body mass index.

†, According to the American Joint Committee on Cancer (AJCC) Thyroid Cancer TNM Staging System (8th ed, 2017).

TABLE S3 Comparison of clinicopathological characteristics among the three patient groups after propensity score matching.

**Variables**	**ISP (n = 50)**		**BAT (n = 50)**		**SAT (n = 50)**		***p***
	**n/mean ± SD/ P_{50} (P_{25}, P_{75})**	**Percent (%)**	**n/mean ± SD/ P_{50} (P_{25}, P_{75})**	**Percent (%)**	**n/mean ± SD/ P_{50} (P_{25}, P_{75})**	**Percent (%)**	
Sex							0.512
Male	10	20.00	13	26.00	15	30.00	
Female	40	80.00	37	74.00	35	70.00	
Age, years	47.50 (43.75,55.00)		47.50 (40.75,53.00)		49.50 (42.50,55.00)		0.576
BMI, kg/m^2	25.63 ± 3.72		25.17 ± 4.42		26.74 ± 3.25		0.111
Preoperative calcium, mmol/L	2.36 (2.31,2.41)		2.36 (2.30,2.41)		2.36 (2.25,2.42)		0.791
Preoperative PTH, pg/mL	47.20 (38.63,60.05)		45.10 (30.40,65.03)		44.75 (34.30,64.20)		0.741
Preoperative 25-OH-D_3, ng/mL	11.25 (8.82,15.25)		13.80 (9.95,16.00)		14.05 (10.28,16.50)		0.141
Tumor location #							0.116
Unilateral	15	36.60	26	55.30	25	56.80	
Bilateral	26	63.40	21	44.70	19	43.20	
Tumor size, mm #	7.00 (4.50,12.50)		8.00 (5.00,12.00)		6.00 (4.00,13.75)		0.613
Multifocality#							0.131
Single	11	26.80	22	46.80	19	43.20	
Multiple (2)	30	73.20	25	53.20	25	56.80	
Number of LND#	8.00 (4.00,10.00)		7.00 (3.00,10.00)		5.00 (2.00,10.00)		0.553
Tumor staging †, #							0.785
Phase 1	38	92.70	45	95.70	27	95.50	
Phase 2	3	7.30	2	4.30	2	4.50	
PTG yield	3.00 (2.00,3.00)		3.00 (2.75,3.00)		3 (3.00,3.00)		0.284

Total of 132 patients with tumors.

BMI, body mass index.

† American Joint Committee on Cancer – Thyroid Cancer TNM Staging System (8th ed, 2017).

TABLE S4 Postoperative HypoPT occurrence among the three patient groups after propensity score matching (n = 50).

**Time**	**ISP (n/%)**	**BAT (n/%)**	**SAT (n/%)**	***p***
POD 1	31/62.00	15/30.00 a	29/58.00 b	0.002
POD 3	25/50.00	11/22.00 a	25/50.00 b	0.004
POW 1	21/42.00	9/18.00 a	21/42.00 b	0.014
POW 2	7/14.00	6/12.00	11/22.00	0.353
POM 1	6/12.00	5/10.00	5/10.00	0.932
POM 3	4/8.00	3/6.00	2/4.00	0.701
POM 6	3/6.00	3/6.00	2/4.00	0.876

a, $p < 0.05$ vs. ISP group; b, $p < 0.05$ vs. BAT group

TABLE S5 Postoperative serum calcium among the three patient groups **(**n = 60**)**.

**Time**	**ISP (**mmol/L**)**	**BAT (**mmol/L**)**	**SAT (**mmol/L**)**	***p***
POD 1	2.29 ± 0.13	2.28 ± 0.11	2.13 ± 0.13	0.918
POD 3	2.16 ± 0.16	2.21 ± 0.15	2.18 ± 0.17	0.325
POW 1	2.23 ± 0.17	2.30(2.26,2.36)	2.29 ± 0.14	0.087
POW 2	2.33 (2.20,2.40)	2.34 (2.28,2.42)	2.32 ± 0.14	0.209
POM 1	2.33 (2.25,2.39)	2.34(2.27,2.42)	2.36(2.20,2.40)	0.548
POM 3	2.28 (2.23,2.39)	2.33 (2.27,2.39)	2.35 (2.23,2.38)	0.223
POM 6	2.28 (2.16,2.39)	2.32 ± 0.12	2.30(2.22,2.43)	0.144

TABLE S6 Postoperative serum calcium among the three patient groups after propensity score matching (n = 50).

**Time**	**ISP** (mmol/L)	**BAT** (mmol/L)	**SAT** (mmol/L)	***p***
POD 1	2.28 ± 0.13	2.28 ± 0.11	2.28(2.17,2.35)	0.625
POD 3	2.16 ± 0.16	2.19 ± 0.16	2.16 ± 0.18	0.414
POW 1	2.21 ± 0.21	2.30(2.20,2.36)	2.27 ± 0.15	0.553
POW 2	2.32(2.20,2.40)	2.32 ± 0.12	2.35(2.20,2.38)	0.324
POM 1	2.30 ± 0.13	2.34(2.26,2.41)	2.33(2.21,2.40)	0.553
POM 3	2.28(2.23,2.38)	2.32 (2.26,2.39)	2.32 (2.23,2.38)	0.387
POM 6	2.27 ± 0.15	2.31 ± 0.13	2.30(2.21,2.43)	0.310

TABLE S7 BAT survival rates at different follow-up time points by survival criteria after propensity score matching **(**n = 50**).**

Time	At 1.5-fold ratio*		At 1.2-fold ratio *		*p*
	Number of survivors (n)	Graft survival rate (%)	Number of survivors (n)	Graft survival rate (%)	
POD 1	0	0.00%	13	26.00%	<0.001
POD 3	0	0.00%	17	34.00%	<0.001
POW 1	14	28.00%	24	48.00%	0.039
POW 2	21	42.00%	25	50.00%	0.422
POM 1	22	44.00%	29	58.00%	0.161
POM 3	23	46.00%	28	56.00%	0.317
POM 6	29	58.00%	37	74.00%	0.091

* PTH concentration ratio between the transplanted and the non-transplanted sides.

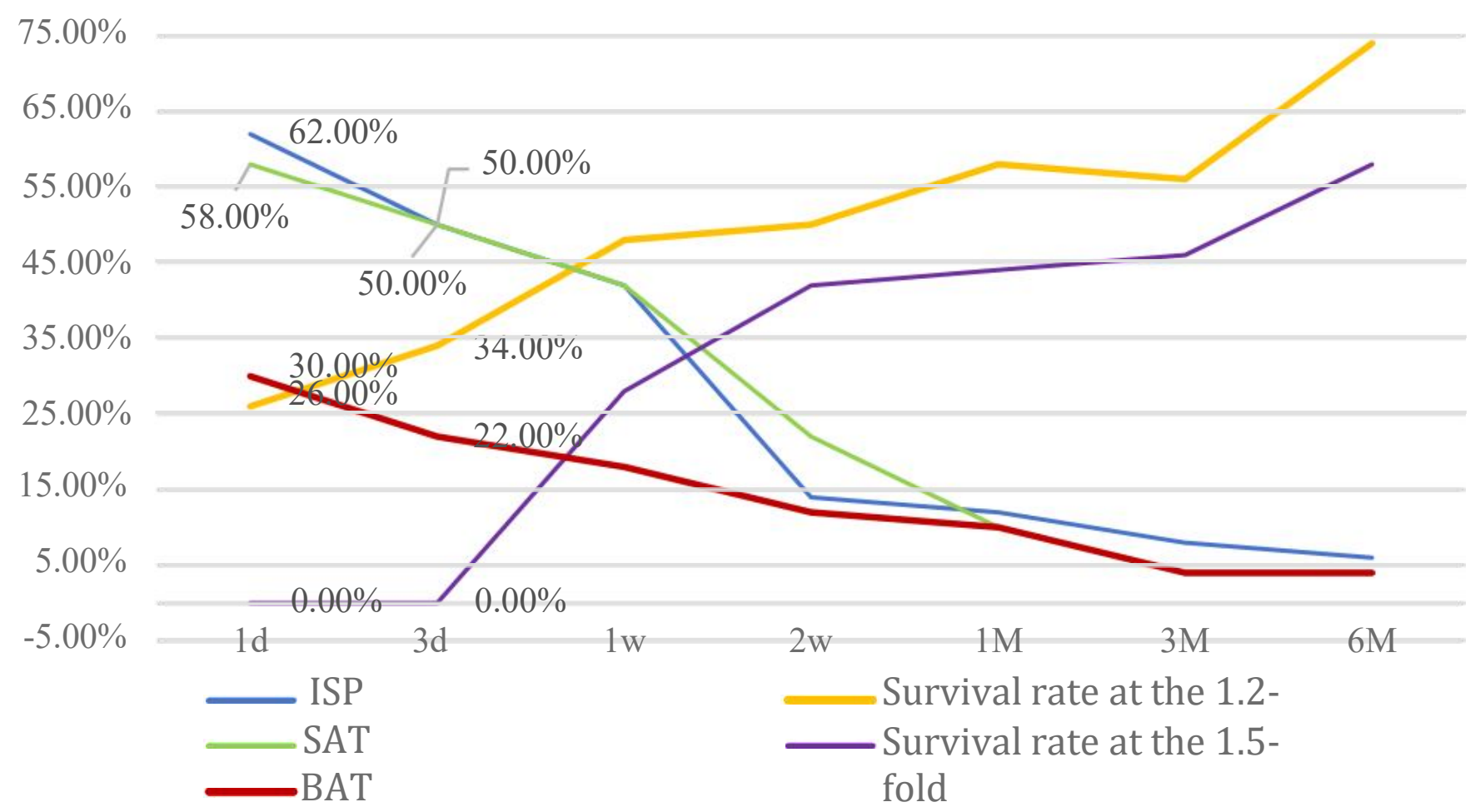

FIGURE S1 Postoperative HypoPT incidence of the three groups and survival rates under the different criteria after propensity score matching **(**n = 50**).**
